# Supplementary material for: Adsorption Mechanism of Patulin from Apple Juice by Inactivated Lactic Acid Bacteria Isolated from Kefir Grains
Source: Toxins (Basel). 2021 Jun 22;13(7):434. doi: 10.3390/toxins13070434 (PMC8309945; doi:10.3390/toxins13070434)
Supplement: Supplementary file 1 [file toxins-13-00434-s001.zip › toxins-1213591-supplementary.pdf]

Supplementary Materials: Adsorption Mechanism of Patulin from Apple Juice by Inactivated Lactic Acid Bacteria Isolated from Kefir Grains

Pascaline Bahati, Xuejun Zeng, Ferdinand Uzizerimana, Ariunsaikhan Tsoggerel, Muhammad Awais, Guo Qi; Rui Cai, Tianli Yue and Yahong Yuan

Results

The target of this study was to determine whether there were other LAB strains isolated from kefir grains additional to those previously reported that can remove patulin from apple juice. The results showed that all of the tested LAB strains were able to remove a high amount of patulin through the adsorption at significant differences, except for LP10 (Figure 1). The high removal amount of patulin was achieved in juice having a 4.6 pH, 15° Brix at 30 °C. The same findings were previously reported by many researchers [1–4]. The results also showed that the toxin reduction was strain- and patulin concentration-dependent [5,6]. This was remarkable for all used bacteria strains in all juices, which sharply increased the adsorption amount, except for LP10, which showed no significant removal between two concentrations. This could be a result of the high concentration of patulin, as well as the composite interaction of several factors. Our experiment outcome showed that no detrimental effects were found on both °Brix and pH level in all treated juices with both concentrations 100 and 200 μg/L by heat-inactivated kefir grain cells, as shown in Figure 1 and Table 2, respectively.
The involved functional groups and adsorption sites of all strains in different juices were identified by FTIR analysis. Figure S3A,B shows the peaks and their corresponding wavenumbers before and after patulin loading in AJE (apple juice with 15 °Brix, pH = 4.6).

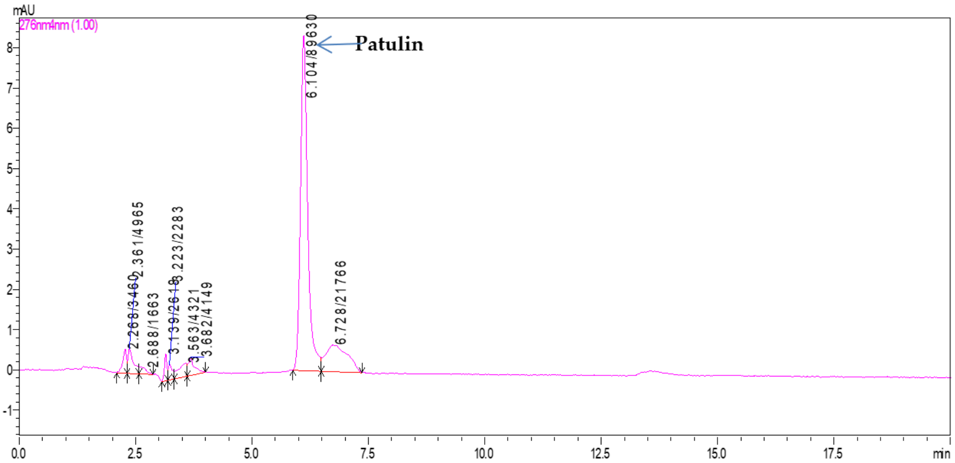

Figure S1. HPLC standard obtained patulin representation. Patulin (1000 μg/L) was reconstituted with 1 ml of deionized water adjusted to a pH of 4.0 with acetic acid. The retention time was approximately 6.1 min, and the wavelength was set at 267 nm.

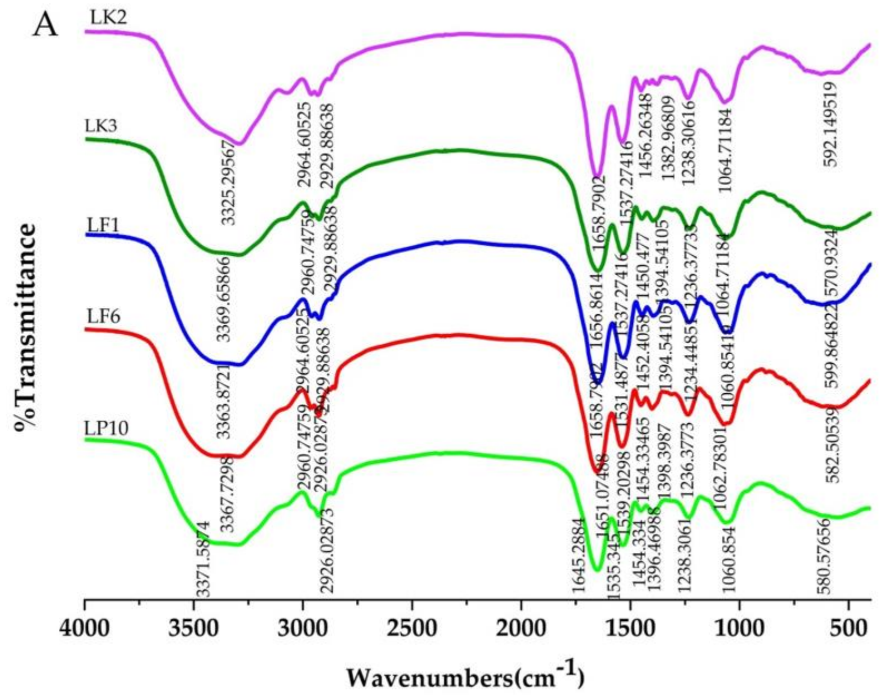

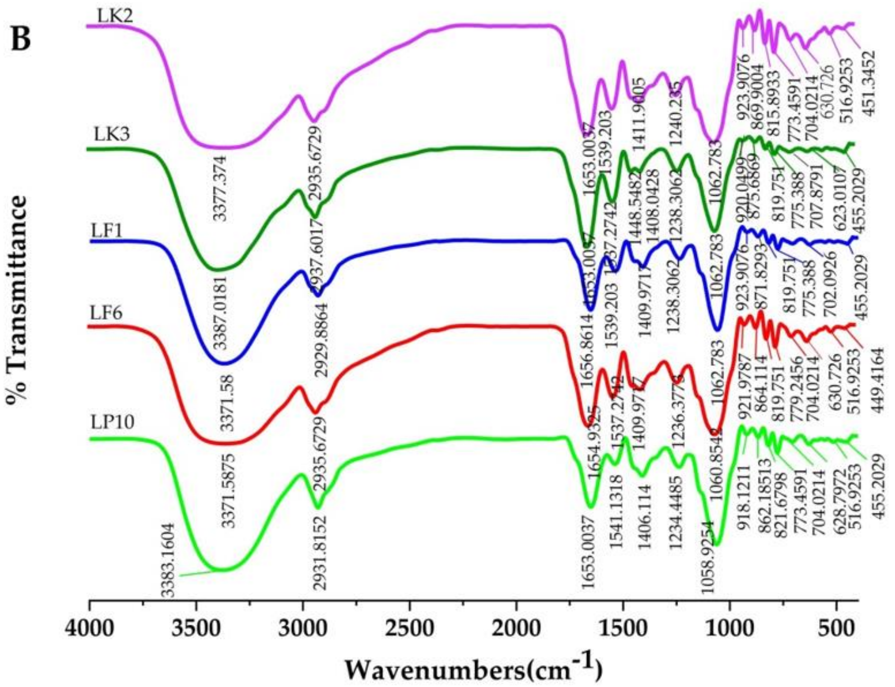

Figure S2. (**a**,**b**) FTIR spectra of heat-inactivated kefir grain cell LAB (LK2, LK3, LF1, LF6, and LP10) before and after loading patulin, respectively. Certain changes in the FTIR spectra between the unexposed and exposed bacterial cells were manifested; some peaks around 2960 cm^{-1} disappeared, some band spectra were shifted, and there was an increase in peak intensity at around 1640 cm^{-1}. Moreover, there were some distinct absorption bonds for aromatic, alkenes, carboxyl acids, and alkyl halides obtained below 1000 cm^{-1}. Nevertheless, the shape of each peak of the sample was held. Thus, the primary morphology of patulin-exposed bacterial cells was not entirely lost.

References

1. Hatab, S.; Yue, T.; Mohamad, O. Reduction of Patulin in Aqueous Solution by Lactic Acid Bacteria. *J. Food Sci.* **2012**, *77*, M238–M241.
2. Hatab, S.; Yue, T.; Mohamad, O. Removal of patulin from apple juice using inactivated lactic acid bacteria. *J. Appl. Microbiol.* **2012**, *112*, 892–899.
3. Sajid, M.; Mehmood, S.; Niu, C.; Yuan, Y.; Yue, T. Effective adsorption of patulin from apple juice by using non-cytotoxic heat-inactivated cells and spores of Alicyclobacillus strains. *Toxins (Basel).* **2018**, *10*, 344.
4. Guo, C.; Guo, M.; Zhang, S.; Qin, D.; Yang, Y.; Li, M. Assessment of patulin adsorption efficacy from aqueous solution by water-insoluble corn flour. *J. Food Saf.* **2018**, *38*, e12397.
5. Wang, L.; Yue, T.; Yuan, Y.; Wang, Z.; Ye, M.; Cai, R. A new insight into the adsorption mechanism of patulin by the heat-inactive lactic acid bacteria cells. *Food Control* **2015**, *50*, 104–110.
6. Wang, L.; Wang, Z.; Yuan, Y.; Cai, R.; Niu, C.; Yue, T. Identification of key factors involved in the biosorption of patulin by inactivated lactic acid bacteria (LAB) cells. *PLoS One* **2015**, *10*. 0143431.
